# Well-Dispersed Silver Nanoparticles on Cellulose Filter Paper for Bacterial Removal

**DOI:** 10.3390/nano11030595

**Published:** 2021-02-27

**Authors:** Hsiu-Wen Chien, Ming-Yen Tsai, Chia-Jung Kuo, Ching-Lo Lin

**Affiliations:** 1 Department of Chemical and Material Engineering, National Kaohsiung University of Science and Technology, Kaohsiung 807618, Taiwan; c108146150@nkust.edu.tw (M.-Y.T.); J109246114@nkust.edu.tw (C.-L.L.); 2 Photo-Sensitive Material Advanced Research and Technology Center (Photo-SMART Center), National Kaohsiung University of Science and Technology, Kaohsiung 807618, Taiwan; k.chiajung@gmail.com

**Keywords:** polydopamine, polyethyleneimine, silver nanoparticle, cellulose, anti-bacteria

## Abstract

In this study, a polydopamine (PDA) and polyethyleneimine (PEI)-assisted approach was developed to generate well-distributed PDA/PEI/silver (PDA/PEI/Ag) nanocomplexes on the surfaces of commercial cellulose filter papers to achieve substantial bacterial reduction under gravity-driven filtration. PDA can bind to cellulose paper and act as a reducer to produce silver nanoparticles (AgNPs), while PEI can react with oxidative dopamine and act as a dispersant to avoid the aggregation of AgNPs. The successful immobilization of PDA/PEI/Ag nanocomplexes was confirmed by scanning electron microscopy (SEM), X-ray diffraction (XRD), and Fourier transform infrared spectroscopy (FTIR). *Staphylococcus aureus* (*S. aureus*) and *Escherichia coli* (*E. coli*) were used as pathogen models to test the efficacy of the PDA/PEI/Ag nanocomplex-incorporated filter papers. The PDA/PEI/Ag nanocomplex-incorporated filter papers provided a substantial bacterial removal of up to 99% by simple gravity filtration. This work may be useful to develop a feasible industrial production process for the integration of biocidal AgNPs into cellulose filter paper and is recommended as a local-condition water-treatment technology to treat microbial-contaminated drinking water.

## 1. Introduction

Mussel adhesive proteins, such as bio-inspired polydopamine (PDA), have attracted extensive interest for surface modifications owing to their suitability in a wide range of coating substrates and ability to further react with amine groups, thiol groups, or other chemical molecules after deposition [1,2,3]. PDA layers are fabricated through the oxidative self-polymerization of dopamine with oxygen under alkaline conditions [4]. The catechols in the PDA layer enable the formation of o-quinones, which can further react with a variety of nucleophiles, such as amine and thiol groups, through Michael addition or Schiff base reaction to form crosslinks [5]. Moreover, catechol is the conjugate acid of chelating agents, which are used widely to coordinate strongly to metals through two adjacent hydroxyls. The formation of metal nanoparticles (NPs) by catechol chemistry has the advantage of forming robust interfaces between metal surfaces and organic molecules compared to other structures [6].

Silver nanoparticles (AgNPs) refer to metallic silver at the nanometer scale. AgNPs can be used in a variety of materials and equipment, such as biomedical, mechanical, electronic, and environmental industries [7,8]. Recently, based on the antibacterial effect of AgNPs, they have been added to the design of many products to make them self-cleaning and antibacterial, thereby achieving a safe and hygienic environment [9]. The “top-down” mechanical grinding method is a common method to prepare AgNPs. The other method is “bottom-up”, which consists of exciting silver ion by the chemical reducing agent or light irradiation to make the silver ion receive electrons to form metallic silver [10]. However, these production methods usually require expensive equipment and toxic reagents hazardous to the environment and living organisms. Thus, there is an urgent need to develop an alternative, cost-effective, safe, and environmentally friendly production process for AgNPs.

Recently, the utilization of PDA layers for the formation of AgNP assemblies has been applied in a wide variety of fields [11,12,13]. For example, Yang et al. coated a thin film of PDA on a polyamide reverse osmosis (RO) membrane for 2 h and subsequently immobilized silver ions in situ for 5 h to form dispersed AgNPs [11]. Similarly, Liu et al. immersed a sericin/agar film into a dopamine solution (pH 8.5) for 12 h and immobilized AgNPs for 4 h through the reduction of silver ions by PDA [13]. The substrates were first dipped into an alkaline-dopamine solution for up to several hours for the deposition of thin adherent PDA layers, followed by the reduction of metal ions. Despite the simplicity of this two-step method for the synthesis of metal NPs, the reported protocols are usually time-consuming.

To apply this method in industrial production, we established a simpler procedure for anchoring AgNPs on a surface. In this study, we developed a one-step method to immobilize AgNPs with the assistance of PDA. By mixing dopamine and silver ions under basic conditions, PDA/Ag nanocomplexes were formed and immobilized on cellulose. However, the distribution of PDA/Ag nanocomplexes on cellulose was very uneven and severely agglomerated. In order to improve such defects, we further modified the synthetic formula, that is, we added polyethyleneimine (PEI) to the mixing solution of dopamine and silver ions. Here, we investigate the effect of the various PEI concentration on the distribution and the morphology of PDA/PEI/Ag nanocomplexes on filter paper. The PDA/PEI/Ag nanocomplex-incorporated cellulose was characterized using scanning electron microscopy (SEM), X-ray diffraction (XRD), and Fourier transform infrared spectroscopy (FTIR). Then, the antimicrobial activity of the silver-modified substrates against *Staphylococcus aureus* (*S. aureus*) and *Escherichia coli* (*E. coli*) was examined. The results confirmed that the cellulose incorporated with PDA/PEI/Ag nanocomplexes through the rapid one-step method has promising applications for bacterial removal.

## 2. Materials and Methods

### 2.1. Materials

Filter papers (Whatman Grade 5 CAT No. 1005-090) with 9 cm diameter and 2.5 μm pore size were employed. Dopamine hydrochloride (99%) was purchased from Acros (Geel, Belgium). Silver nitrate (AgNO_3_) and branched PEI (Branched, MW 10,000 Da) were purchased from Alfa Aesar (Ward Hill, MA, USA). All other chemicals were purchased from Sigma-Aldrich (St. Louis, MO, USA) unless stated otherwise.

*S. aureus* (ATCC 21351) and *E. coli* (ATCC 23501) were obtained from the Food Industry Research and Development Institute (Hsinchu, Taiwan). Trypticase soy agar contained 15 g/L agar (BD, Franklin Lakes, NJ, USA) with 15 g/L of tryptone (Cyrusbioscience, New Taipei, Taiwan), 5 g/L of soy peptone (Cyrusbioscience, New Taipei, Taiwan), and 5 g/L of NaCl. The bacterial suspension buffer was prepared by using 0.85% NaCl at pH 7.

### 2.2. Preparation of PDA/PEI/Ag Nanocomplex-Incorporated Filter Papers

Filter papers were directly soaked in a reaction solution consisting of dopamine (2 mg/mL), AgNO_3_ (5 mM) and PEI (0, 0.1, 1, and 10 mg/mL) in tris buffer solution (pH = 8.5, 10 mM). After a two-hour incubation at 25 °C, the filter papers were removed from the solution and rinsed with deionized water to obtain the PDA/PEI/Ag nanocomplex-decorated filter papers; refer to Table 1. Filter papers soaked in 2 mg/mL of dopamine in Tris buffer solution (pH = 8.5, 10 mM) were used as control (hereinafter referred to as PDA filter papers).

### 2.3. Characterization

The morphology of the PDA/PEI/Ag nanocomplex-decorated filter papers was examined using field emission SEM (FESEM, JEOL JSM-6701F, Tokyo, Japan). An XRD analysis in scanning mode was carried out with a high-resolution X-ray diffractometer (Bede D1, Durham, UK) to study the crystalline nature of the papers. The process was operated at 40 kV and with a current of 40 mA with Cu/K_α_ radiation (λ = 1.5405 Å) in the range of 10–80° in 2*θ* angles with a scanning speed of 0.05°·s^−1^. The surface chemical structures were detected with an FTIR spectrometer (Spectrum One, PerkinElmer, Waltham, MA, USA) with an attenuated total reflection (ATR) diamond crystal accessory; the spectra were obtained in absorbance mode from 4000 to 650 cm^−1^ with 48 scans at 4 cm^−1^ resolution.

### 2.4. Evaluation of Antimicrobial Activity

The antibacterial characteristics of the PDA/PEI/Ag nanocomplex-decorated filter papers were tested against *S. aureus* and *E. coli* using the inhibition zone method. First, the nutrient agar medium was sterilized by autoclaving, and then the bacterial suspension of *S. aureus* and *E. coli* (approximately 10^6^CFU/mL) was evenly spread on the medium by the plate coating method, and a round filter paper with a diameter of 7 mm was carefully placed on top of the agar plates. After overnight incubation at 37 °C, the diameter of the inhibition zone was measured to evaluate the antibacterial effect [14].

The antibacterial effect was also investigated in dynamic contact conditions. The test samples (3 × 3 cm^2^) were immersed in 25 mL of suspension on a rotary shaker (100 rpm) at room temperature with different contact times. At the appropriate time, the optical density (OD, 600 nm) of the bacterial solution was measured. Each sample was measured in triplicate.

### 2.5. Flux

The water flux through the paper was measured with a simple gravity filtration setup. Briefly, a piece of filter paper (9 cm in diameter) was placed in a Büchner funnel and was held by a clamp. The filtration experiments were performed at a water head of 1, 2 and 4 cm. The water flux was calculated using the equation *J* = *Q*/*AT*, where *J* is the permeation flux (L/m^2^·h), *Q* is the permeation volume of the testing solution (L), *A* is the effective area of the tested membrane (m^2^), and *T* is the time (h) taken for 15 mL of water to permeate.

### 2.6. Bacterial Filtration Efficiency

The filtration efficiency of the PDA/PEI/Ag nanocomplex-decorated filter papers was tested against *S. aureus* and *E. coli* suspensions as contaminated water models. The bacterial suspension of *S. aureus* and *E. coli* (approximately 10^6^ CFU/mL) at an initial water head of 2 cm flowed through each paper. The filtrates were collected, the serial dilutions of the solutions were prepared, and 100 μL of dilutions were spread on sterile agar plates. After incubation at 37 °C for 24 h, the number of colonies in each filtrate was calculated. The bacterial filtration efficiency of the filter paper specimens was calculated using the equation (*A* − *B*)/*A* × 100, where *A* is the number of viable cells (colony-forming unit) in the feeding solution and *B* is the number of viable cells in the filtrate [15,16]. Each sample was examined in triplicate, and the typical images were used.

### 2.7. Silver Release

Silver leaching was examined by filtering deionized water. Briefly, a piece of the PDA/PEI/Ag nanocomplex-decorated filter paper (9 cm in diameter) was placed in a Büchner funnel and was held by a clamp. The water head of 1 cm was applied to the filtration. The filtrate was collected and further passed through a 0.22 μm polyethersulfone (PES) filter. After that, the filtrate was examined for silver content with an inductively coupled plasma optical emission spectrometer (ICP-OES, Thermo Scientific iCAP 7000, Waltham, MA, USA).

### 2.8. Statistical Analysis

The data are reported as means ± standard deviation (SD). The statistical analyses of different groups were performed using the Student’s t-test. Probabilities *p* ≤ 0.05 were considered statistically significant. All statistical analyses were performed using the GraphPad Instat 3.0 program (GraphPad Software, San Diego, CA, USA) [17].

## 3. Results and Discussion

### 3.1. Effect of PEI Concentration on the Formation of Nanocomplexes

**Figure 1:**
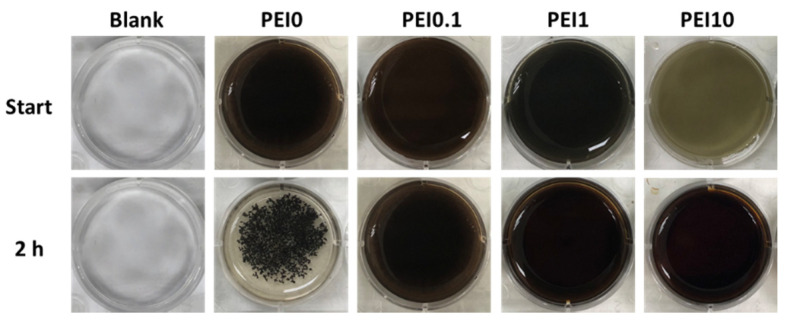
Photographs of the color of the reaction solution.

**Figure 2:**
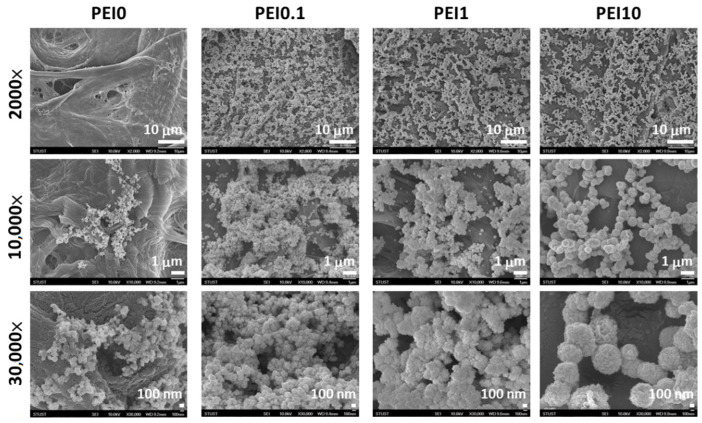
SEM views of the different nanocomplexes on filter papers.

First, we observed the effect of various PEI concentrations on the color of the reaction solution. As shown in Figure 1, when dopamine, PEI, and AgNO_3_ mixed into the tris buffer solution, the color of the mixture immediately became a gray-black color, which means that this was a rapid reaction. Notably, the color faded as the PEI concentration increased to 10 mg/mL, implying that excess PEI probably inhibits the initial complex formation. However, the PEI10 solution could quickly turn gray-black within 3 min. After 2 h of reaction, we found that the solution without PEI (PEI0) had insoluble black aggregates, while the color of the solution containing PEI (including PEI0.1, PEI1, and PEI10) remained saturated gray-black. Next, we investigated the morphologies of nanocomplexes by using SEM. As shown in Figure 2, PEI0 had many irregular aggregations on the surface of the filter papers, while for PEI0.1, PEI1, and PEI10, the nanocomplexes on the surface of the filter papers were uniformly distributed. Notably, when the concentration of PEI in the solution was increased, the nanocomplexes began to assemble. As shown in magnified images (30,000×), the nanocomplexes of PEI0.1 were uniform in size with an average diameter of approximately 100 nm, while PEI10 was composed of multiple nanocomplexes assembled into an approximately spherical shape with a mean diameter of 1 μm.

**Figure 3:**
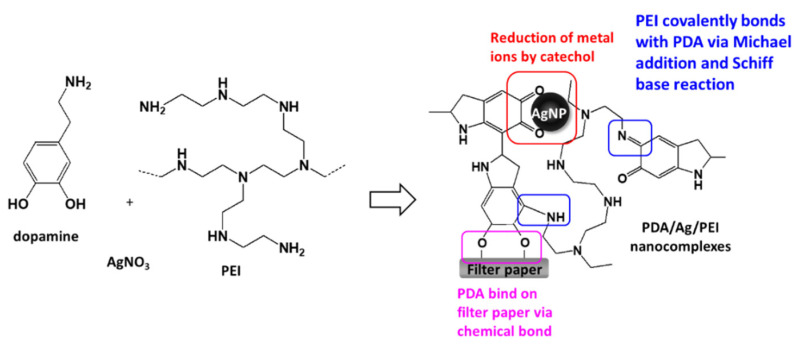
Schematic illustration and possible mechanism of the loading of PDA/PEI/Ag nanocomplexes onto cellulose filter paper.

A previous study indicated that when dopamine and silver ions were mixed together, a core-shell nanostructure was formed, that is, the core of AgNPs and the surrounding PDA layer [18]. Because PDA technology has inherent melanin aggregation properties [19], it was easy to form aggregates with different sizes. The reason could be that the nanocomplexes of PEI0 were irregular agglomerations. Herein, we aim to add PEI to prevent the aggregation phenomenon of nanocomplexes and anchor the nanocomplexes on filter paper. Previous literature indicated that PEI could form covalent bonds with oxidized dopamine via the Michael addition and Schiff base reaction [20]. Since the adhesion molecules (i.e., catechol groups) of PDAs were surrounded by PEI, the aggregation between PDAs was avoided [20]. This could explain why the nanocomplexes of PEI0.1 were well dispersed. On the other hand, amines of PEI are capable of forming complexes with metal ions via coordination [21,22]. However, high concentrations of PEI in aqueous solution probably change the conformation to a globular structure due to polymer chain entanglement [21], which in turn leads to the self-assembly of the nanocomposite into a spherical shape (such as PEI10). In addition, when the cellulose papers were immersed into a dopamine aqueous solution, the catechol groups of dopamine tended to react with the hydroxyl groups, and stable chemical bonds were formed between the cellulose and catechol [23]. Then, the assembly eventually formed an adherent PDA stacking layer on the surface of cellulose [23]. Therefore, we can speculate that the formation mechanism of PDA/PEI/Ag nanocomplexes on filter paper were as shown in Figure 3.

### 3.2. Characterization of Nanocomplexes

**Figure 4:**
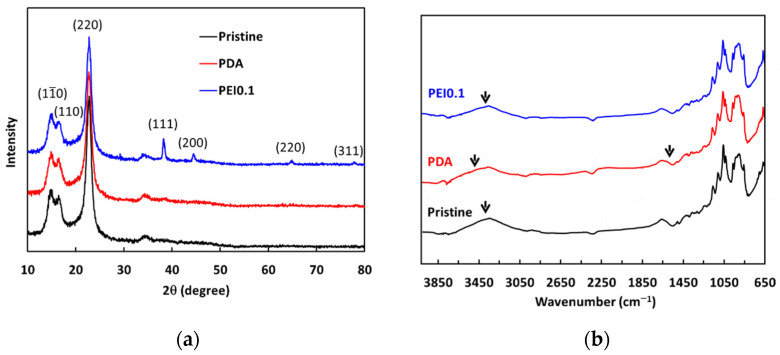
(**a**) XRD spectra and (**b**) ATR-FITR spectra of as-prepared filter papers.

From the above results, we found that the nanocomplexes of PEI0.1 had the best dispersibility. Therefore, we further analyzed XRD patterns to understand more detailed structural information (Figure 4a). For pristine cellulose papers, the characteristic peaks at 2*θ* = 14.7°, 16.8°, and 22.8° correspond to the (1$\bar{1}$0), (110), and (220) crystallographic planes of the monolithic cellulose type I, respectively [24]. The XRD patterns of the PDA samples are not significantly different from those of the pristine filter papers. In the XRD patterns of the PEI0.1 samples, there are four extra characteristic peaks of silver at 2*θ* = 38°, 44°, 64°, and 77°, corresponding to the (111), (200), (220), and (311) planes, respectively. The peaks of the PEI0.1 filter papers matched well with those of the face-centered cubic crystal structure of silver (JCPDS No. 65-2871) [25]. The results verify the successful immobilization of PDA/PEI/Ag nanocomplexes on the filter papers as a result of the reductive effect of the catechol groups.

The ATR-FTIR analysis further verified the existence of PDA/PEI/Ag complexes on the surface of the filter papers. The pristine cellulose paper spectrum shown in Figure 4b shows a strong, broad band from 3600–3000 cm^−1^ resulting from the stretching vibrations of the O-H groups, while the band at 2877 cm^−1^ is the result of the asymmetric and symmetric stretching of the C-H bonds in the CH_2_OH groups. The peaks between 1400 and 900 cm^−1^ indicate deformation, bending, or stretching vibrations of several types of bonds, including C-H and C-O. The FTIR spectrum of the PDA filter paper also shows peaks similar to those of the pristine filter paper spectrum. However, the broad band shifted from 3314 to 3425 cm^−1^, indicating the formation of N-H groups due to the deposition of PDA. In addition, a new weak band at 1610 cm^−1^ was observed, which is attributed to the benzyl functional groups in the PDA [26]. Between the spectra of the PDA and PEI0.1 filter papers, the broad band shifted from 3314 to 3301 cm^−1^, indicating that the O-H groups may be responsible for the reduction of silver ions to AgNPs [18]. These results further confirm that the filter papers were successfully incorporated with PDA/PEI/Ag nanocomplexes.

### 3.3. Antibacterial Activity

**Figure 5:**
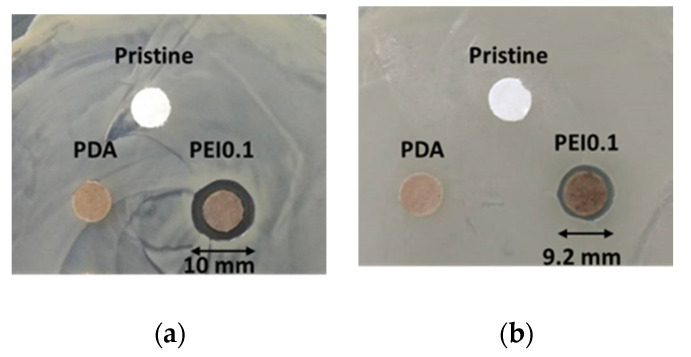
Antibacterial activity of diffusion inhibition zone test for different filter papers. (**a**) *S. aureus* (**b**) *E. coli.*

Cellulose paper is a material commonly used in our daily lives. It is a flexible and versatile material with many simple applications, but it is unsuitable as a substrate for anti-bacterial materials. In this study, we used cellulose paper to covalently modify a paper substrate with PDA/PEI/Ag nanocomplexes so that it obtained antibacterial functions. The antibacterial activity of the PEI0.1 filter papers against Gram-positive *S. aureus* and Gram-negative *E. coli* was first evaluated using an inhibition zone method. Figure 5a,b show images of the *S. aureus* and *E. coli* after the inhibition, respectively, using the pristine, PDA, and PEI0.1 filter papers. It can be seen from the figure that the pristine and PDA filter papers have no obvious inhibition zones, indicating no antibacterial activity. In contrast, the PEI0.1 filter papers show inhibition zones with diameters of approximately 10.0 and 9.2 mm on *S. aureus* and *E. coli*, respectively. This indicates that the PEI0.1 filter paper has a broad-spectrum antibacterial ability due to the effect of the deposited PDA/PEI/Ag nanocomplexes.

**Figure 6:**
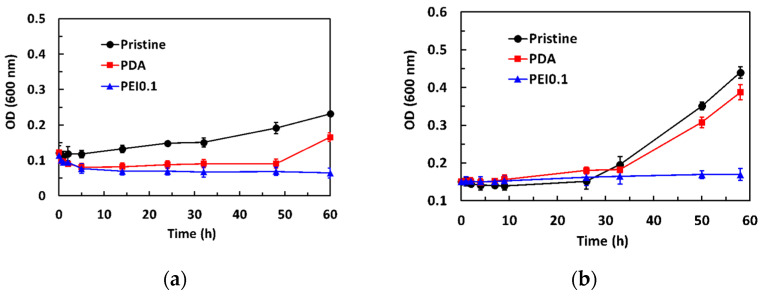
Antibacterial activity of dynamic contact test for different filter papers. (**a**) *S. aureus* (**b**) *E. coli.*

The dynamic antibacterial activity of the PEI0.1 filter papers against *S. aureus* and *E. coli* was also evaluated, using a dynamic contact test. The OD_600_ values of the starting concentration on *S. aureus* and *E. coli* were 0.12 and 0.15, respectively. Figure 6a shows that the OD_600_ values of *S. aureus* in the pristine group gradually increased with increasing contact time. However, the OD_600_ values in the PDA group decreased to 0.09 within the first 5 h of contact and then slightly increased. After 50 h of contact, the OD_600_ values increased significantly. Compared to the pristine and PDA filter papers, the introduction of PDA/PEI/Ag nanocomplexes into the filter papers did not show any obvious increase in the number of viable bacteria, even for the longest contact time, indicating that the PEI0.1 filter papers could effectively inhibit bacterial growth. As expected, the OD_600_ values of *E. coli* in the pristine and PDA group slightly increased with increasing contact time and rapidly increased after 24 h of contact (Figure 6b). However, the OD_600_ values remained constant, indicating that the PEI0.1 filter papers had a biocidal ability.

Previous studies have reported that PDA coatings have a moderate antibacterial effect [27,28]. In our work, using a dynamic contact test, we observed a similar antimicrobial activity of PDA against Gram-positive *S. aureus* but not against Gram-negative *E. coli*. The difference in the antibacterial effect between Gram-positive and Gram-negative bacteria might be related to their cell wall composition. Gram-positive bacteria have cell walls composed of peptidoglycan, while Gram-negative bacteria have an outer membrane composed of lipopolysaccharides in addition to cell walls composed of peptidoglycan [29]. The outer lipopolysaccharide layer of Gram-negative bacteria probably prevented PDA from passing through the cell wall. Thus, the antibacterial efficacy of PDA was better against *S. aureus* than *E. coli*. Although *S. aureus* and *E. coli* were not eliminated by the PDA, the growth of *S. aureus* and *E. coli* could be effectively inhibited by the developed PEI0.1 filter papers.

### 3.4. Application of Bacterial Removal

**Figure 7:**
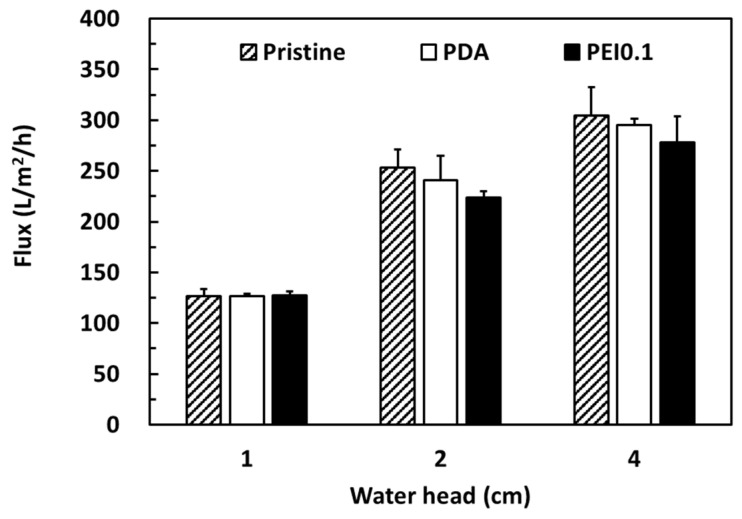
Pure water fluxes of different filter papers.

Cellulose filter papers are widely used in gravity filtration and are separated by trapping the particles in a random matrix of cellulosic fibers. Here, we attempted to use the PDA/Ag/PEI nanocomplex-incorporated cellulose papers as filter membranes for bacterial removal. According to previous works, PDA-modified membranes have a lower flux and longer filtration cycle than other membranes because their denser PDA coating blocks the porous fabric matrix [30,31]. To confirm that the PDA/PEI/Ag coating did not seriously reduce the filtration ability, the water flux of different coating filter papers was investigated. Figure 7 shows a comparison of the pure water flux performances of the pristine, PDA, and PEI0.1 filter papers under gravity-driven conditions with a water head of 1, 2, and 4 cm. As expected, the water flux (approximately 300 L/m^2^/h) was the highest at a water head of 4 cm and the lowest (approximately 125 L/m^2^/h) at a water head of 1 cm. Under various water head conditions, the water flux of the pristine, PDA, and PEI0.1 filter papers was similar. Despite the PDA/PEI/Ag nanocomplexes anchored on the surface, probably blocking the pores to some extent, their water flux under various water heads was similar to the corresponding value of the pristine filter papers. These results suggest that the permeability of the PEI0.1 filter paper was sufficiently high for gravity filtration.

**Figure 8:**
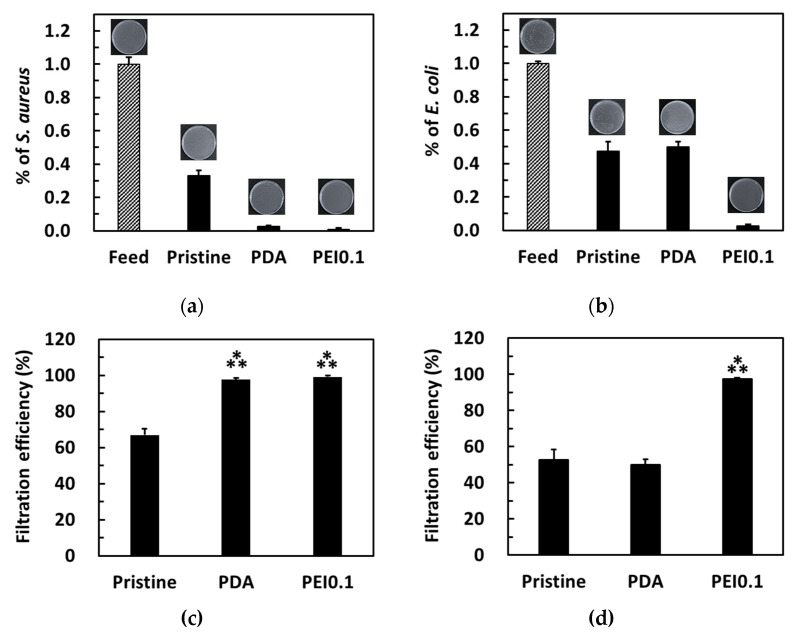
Colonies formed on the agar plate by *S. aureus* (**a**,**c**) and *E. coli* (**b**,**d**) in water before and after filtration through the different filter papers. Filtration efficiency for different filter papers. ***, *p* < 0.001 vs. pristine.

The bacterial filtration efficiency was further tested using a simple gravity-driven filtration experiment. After filtration, some bacteria were detected in the filtrates of the pristine and PDA filter papers, while no bacteria were detected in the filtrates of the PEI0.1 filter papers (Figure 8a,b). For *S. aureus*, the pristine, PDA, and Ag filter papers provided a filtration ability of approximately 67%, 97.6%, and 99.5%, respectively (Figure 8c). For *E. coli*, both pristine and PDA filter papers provided a filtration ability of approximately 50% (Figure 8d). Notably, only 2.7% of *E. coli* was found in the filtrate of the PEI0.1 filter papers, indicating an excellent filtration efficiency. Because the pristine filter papers did not show any AgNP loading and bactericidal effect, the filtration efficiency can be attributed to physical removal [32]. Compared to the pristine filter papers, the PEI0.1 filter papers provide an additional antibacterial filtration efficiency because of the silver’s biocidal efficacy.

In the past, a combination of silver-based systems and porous plastic membranes was among the most common structures for contaminant removal and microorganism inactivation [33,34,35,36,37,38]. The silver disinfection is believed to be a result of silver atoms binding to thiol groups in enzymes, subsequently resulting in enzymatic deactivation [39]. Another possible theory is that silver ions enter the cell and denature the DNA [39,40]. However, silver is potentially toxic to humans at high doses [41,42]. Thus, we measured the concentration of silver in the filtrate to assess potential health risks. The leached silver concentration in the filtrate of the PEI0.1 filter papers was found to be less than 10 ppb, which is far below the 100 ppb limit considered safe for drinking water according to the World Health Organization’s (WHO’s) guidelines. The guidelines state that, where silver salts are used to maintain the bacteriological quality of drinking water, levels of silver up to 100 μg/L can be present without risk to human health [43].

In this text, we employed cellulose papers as filter membranes to load PDA/PEI/Ag nanocomplexes via a one-step mussel-inspired coating for bacterial removal. Because of the negative impact of plastic materials on the environment, an increasing number of research studies utilize cellulose fibers or other lignocellulosic materials as green alternatives [44,45,46]. Cellulose is the skeletal component of plants and is biodegradable and eventually recyclable. After utilization and burial, it gradually biodegrades in soil. Therefore, cellulose can be regarded as an environmentally friendly material with low pollution effects. In addition, cellulose-based filter papers have been fabricated on a large scale by a facile and low-cost papermaking technology. Thus, cellulose paper can be a good alternative to plastic membranes. On the other hand, the generation of PDA/PEI/Ag nanocomplexes in our study by the reduction–oxidation potential of catechol can be regarded as an eco-friendly method. Compared to the two-step or multistep procedures used in previous studies [47,48], our one-step method provides a more efficient process for immobilizing AgNPs on surfaces. For example, Islam et al. developed a triple-step method to prepare an antimicrobial cellulose paper. First, they functionalized the cellulose paper with succinic acid and then reacted with dopamine to give a dopamine-modified paper. Finally, the dopamine molecules possess strong coordination with silver ions to form the deposition of well-dispersed AgNPs on the paper [47]. However, the reported protocols are time-consuming. Overall, this low-cost, simple, efficient, and environmentally friendly approach and materials are promising for application in rapid bacterial removal to resolve drinking-water problems, especially countries that are poor and lacking in electricity [43].

## 4. Conclusions

We successfully immobilized PDA/PEI/Ag nanocomplexes with good dispersion on cellulose filter papers via a one-step coating of dopamine with silver ions and PEI. The PEI concentration was significantly affected on the distribution and the morphology of PDA/PEI/Ag nanocomplexs. When the reaction composition without PEI, the nanocomplexes were irregular agglomerations. Low PEI concentration could stabilize the nanocomplexes against aggregation, while high PEI concentration caused spherical self-assembly of nanocomplexes. The well-dispersed PDA/PEI/Ag nanocomplexes on the filter papers offered a high antimicrobial activity against both Gram-positive *S. aureus* and Gram-negative *E. coli* via antibacterial assays in the inhibition zone and a dynamic contact test. Furthermore, the PDA/PEI/Ag nanocomplex-incorporated filter papers removed the number of *S. aureus* and *E. coli* by up to ~99% through a simple gravity filtration process. The leaching of silver was less than 10 ppb, which is far below the WHO’s drinking water standard limit of 100 ppb. The green and low-cost PDA/PEI/Ag nanocomplex-incorporated filter papers enable their use in large-scale industrial productions and are promising candidates as bacterial removal for local condition water-treatment technology.

## Figures and Tables

**Table 1:** The composition of the reaction solution.

Filter Papers	Dopamine (mg/mL)	AgNO_3_ (mM)	PEI (mg/mL)
PDA	2	0	0
PEI0	2	5	0
PEI0.1	2	5	0.1
PEI1	2	5	1
PEI10	2	5	10
